# Complete genome sequence of *Lysobacter firmicutimachus* SR10, a potential biocontrol agent isolated from the rhizosphere of Chinese chive

**DOI:** 10.1128/mra.01247-25

**Published:** 2026-01-12

**Authors:** Yingtong Xu, Shanren Li

**Affiliations:** 1 National and Local United Engineering Research Center of Industrial Microbiology and Fermentation Technology, College of Life Sciences, Fujian Normal University 12425, Fuzhou, China; University of Strathclyde, Glasgow, United Kingdom

**Keywords:** *Lysobacter firmicutimachus*, complete genome, biological control

## Abstract

We report the complete genome sequence of *Lysobacter firmicutimachus* SR10, a potential biocontrol agent isolated from the rhizosphere of Chinese chive. The assembled genome comprises a single circular chromosome of 5,355,594 bp with a GC content of 68.98%, providing genetic insights into its potential antimicrobial activity.

## ANNOUNCEMENT

*Lysobacter* is a genus of gram-negative bacteria recognized as effective biocontrol agents for their production of diverse antibiotic natural products (1). *Lysobacter firmicutimachus* SR10 was isolated in July 2019 from the rhizosphere soil of Chinese chive in Jianou City, China. For isolation, approximately 5 g of soil collected from the root systems of multiple plants was suspended in 50 mL of sterile saline (0.9% NaCl), homogenized by shaking at 200 rpm for 30 min, serially diluted, and plated onto R2A agar supplemented with kanamycin (50 µg/mL). After incubation at 30°C for 48 h, distinct colonies were purified on fresh R2A agar. Here, we report the complete genome sequence of *L. firmicutimachus* SR10 to facilitate the elucidation of the genetic basis underlying its biocontrol potential.

A single colony was inoculated into 50 mL of LB broth and cultured at 30°C with shaking at 200 rpm for 20 h. Genomic DNA was extracted using a Bacterial Genomic DNA Extraction Kit (Tiangen, China) and quantified using both a NanoDrop One spectrophotometer and a Qubit 4.0 fluorometer (Thermo Fisher Scientific, USA). Whole-genome sequencing was performed on the Illumina NovaSeq 6000 and Oxford Nanopore PromethION 48 platforms at Biomarker Technology Co., Ltd. (Beijing, China). For Illumina sequencing, a short-read library was constructed using the Nextera XT DNA Library Preparation Kit (Illumina, USA), yielding 7,830,102 read pairs (total 2.3 Gb). The resulting reads were quality-checked with FastQC v0.11.9 (2) and processed using Trimmomatic v0.33 (3). For Nanopore sequencing, a long-read library was prepared using the SQK-LSK109 Ligation Sequencing Kit without prior shearing or size selection. Basecalling was performed with Guppy v3.2.4 in high-accuracy mode, generating 53,503 raw reads (average length = 12,702 bp; *N*_50_ = 20,316 bp). These raw reads were then filtered using Filtlong v0.2.0 (4) to remove short (<2,000 bp) and low-quality reads (*Q* < 6), resulting in 43,687 filtered reads (average length = 14,309 bp; *N*_50_ = 20,344 bp), which provided approximately 116-fold genome coverage. The filtered long reads were assembled using Canu v1.5 (5), and the resulting contig was circularized using Circlator v1.5.5 (6). The assembly was subsequently polished with Illumina short reads using Pilon v1.22 (7). Gene prediction and annotation were performed with the NCBI Prokaryotic Genome Annotation Pipeline (PGAP v6.7) (8). Unless otherwise noted, default parameters were used for all software.

The complete genome of *L. firmicutimachus* SR10 comprises a single circular chromosome of 5,355,594 bp with a GC content of 68.98%. Genome annotation identified 4,652 genes, including 4,533 protein-coding sequences, 65 RNA genes (6 rRNAs, 55 tRNAs, and 4 noncoding RNAs), and 54 pseudogenes. Species identification was confirmed by average nucleotide identity analysis using OrthoANIu (9), which showed 98.67% identity to the type strain *L. firmicutimachus* PB-6250^T^ (GCF_037027445.1), the value significantly exceeding the 95% species delineation threshold (10). Furthermore, antiSMASH v8.0.4 (11) analysis predicted 12 biosynthetic gene clusters, including putative clusters with homology to those responsible for the biosynthesis of the heat-stable antifungal factor and empedopeptin (Table 1). These results provide a genetic basis for the potential antimicrobial activity of SR10 and support its promise as a biocontrol agent.

**TABLE 1:** Predicted biosynthetic gene clusters in *Lysobacter firmicutimachus* SR10^*a*^

Region	Location (bp)	Type	Most similar known cluster	Similarity confidence
1	1,776,833–1,818,032	Arylpolyene	Xanthomonadin	Medium
2	1,843,016–1,911,482	NRPS	–	–
3	2,368,157–2,388,879	CDPS	–	–
4	2,843,078–2,865,355	Redox-cofactor	–	–
5	3,114,177–3,125,019	RiPP-like	–	–
6	3,412,808–3,456,638	NRPS	–	–
7	3,626,512–3,649,346	Lanthipeptide	–	–
8	4,000,378–4,011,235	RiPP-like	–	–
9	4,026,469–4,094,375	NRPS	Empedopeptin	Low
10	4,705,392–4,754,892	T1PKS, NRPS	Heat-stable antifungal factor	High
11	4,818,933–4,829,790	RiPP-like	–	–
12	4,874,417–4,895,313	Terpene-precursor	–	–

^*a*^ Locations refer to the complete genome sequence of *Lysobacter firmicutimachus* SR10 deposited in GenBank under accession number CP159925. “–” indicates that the information is not available.

## Data Availability

The complete genome sequence of *L. firmicutimachus* SR10 has been deposited in GenBank under accession number CP159925. The associated data are available under BioProject PRJNA1127280 and BioSample SAMN41999030. The Raw Illumina and Nanopore sequencing data have been submitted to the SRA under accession numbers SRR35892036 and SRR36003919, respectively. The complete antiSMASH v8.0.4 output for this genome is available in the Zenodo repository at https://doi.org/10.5281/zenodo.17725326.
